# Supplementary material for: The association between pre-pregnancy body mass index and perinatal death and the role of gestational age at delivery
Source: PLoS One. 2022 Mar 23;17(3):e0264565. doi: 10.1371/journal.pone.0264565 (PMC8942230; doi:10.1371/journal.pone.0264565)
Supplement: S1 Table — (DOCX) [file pone.0264565.s002.docx]

S1 Table: ICD-10 codes definitions of relevant variables.

| **Variable** | **ICD Codes** |
| --- | --- |
| Pre-pregnancy hypertension | O10, O11 |
| Diabetes mellitus | E10,E11, O24.5, O24.6, O24.7 |
| Chronic cardiac disease | I05-I09, I34-I37, I39.1-I39.4, I25, Q20-26, I27.0, I27.2, I27.8, I27.9 |
| Chonic renal disease | N02.2, N03-N05, N08, N18, N25 |
| Chronic hepatic disease | K70-K77, B18 |
| Systemtic lupus | M32 |
| Ashtma | J44, J45 |
| Gestational hypertension | O24.8 |
| Gestational diabetes | O13 |
| Placental previa | O44 |
| Placental disorders | O43, O43.1, O43.2, O43.8, O43.9 |
| Pre-eclampsia | O141,O14.3-O14.9, O11 |
| Congenital anomaly | Q^^ |
