# Supplementary material for: The association between pre-pregnancy body mass index and perinatal death and the role of gestational age at delivery
Source: PLoS One. 2022 Mar 23;17(3):e0264565. doi: 10.1371/journal.pone.0264565 (PMC8942230; doi:10.1371/journal.pone.0264565)
Supplement: S2 Table — Estimates for underweight estimates are not presented as the total effects were not significant. The e-value is also not estimated when the effect is not significant. (DOCX) [file pone.0264565.s003.docx]

S2 Table: The amount of unmeasured confounding (as a risk ratio) between mediator and outcome needed to explain away total, direct and indirect effects. Estimates for underweight estimates are not presented as the total effects were not significant. The e-value is also not estimated when the effect is not significant.

| **BMI Category** | **Overweight** | | **Obese** | |
| --- | --- | --- | --- | --- |
|  | E-value point estimate | E-value CI lower bound | E-value point estimate | E-value CI lower bound |
| Total effect | 1.77 | 1.43 | 2.59 | 2.18 |
| Natural direct | - | - | 1.54 | 1.17 |
| Natural indirect | 1.52 | 1.34 | 2.16 | 1.95 |
| **Adjusted*** |  |  |  |  |
| Total effect | 1.74 | 1.39 | 2.48 | 2.06 |
| Natural direct | - | - | 1.63 | 1.28 |
| Natural indirect | 1.44 | 1.25 | 1.97 | 1.75 |
| **Adjusted**** |  |  |  |  |
| Total effect | 1.73 | 1.39 | 2.47 | 2.05 |
| Natural direct | 1.45 | 1.05 | 1.65 | 1.29 |
| Natural indirect | 1.44 | 1.24 | 1.94 | 1.73 |

| * adjusted for chronic hypertension, smoking, substance/alcohol use, prior stillbirth, prior preterm birth, parity, maternal age,  year of birth, chronic diseases, asthma |
| --- |
| ** adjusted also for pre-pregnancy diabetes, congenital anomalies |
