# Supplementary material for: The association between pre-pregnancy body mass index and perinatal death and the role of gestational age at delivery
Source: PLoS One. 2022 Mar 23;17(3):e0264565. doi: 10.1371/journal.pone.0264565 (PMC8942230; doi:10.1371/journal.pone.0264565)
Supplement: S3 Table — (DOCX) [file pone.0264565.s004.docx]

S3 Table: Comparison of women with and without complete BMI data

|  | Complete BMI  N = 392,820  Number (%) | Missing BMI  N= 153,855  Number (%) | Standardized mean difference |
| --- | --- | --- | --- |
| Maternal age (years) |  |  | 0.083 |
| <20 | 8899 (2.3) | 5583 (3.6) |  |
| 20-34 | 292618 (74.5) | 112623 (73.2) |  |
| 35-39 | 74903 (19.1) | 28686 (18.6) |  |
| ≥40 | 16400 (4.2) | 6963 (4.5) |  |
| Year of delivery |  |  | 0.214 |
| 2004-2006 | 75897 (19.3) | 33191 (21.6) |  |
| 2007-2009 | 84315 (21.5) | 43773 (28.5) |  |
| 2010-2012 | 92633 (23.6) | 34983 (22.7) |  |
| 2012-2014 | 64899 (16.5) | 20183 (13.1) |  |
| 2015-2017 | 75076 (19.1) | 21725 (14.1) |  |
| Male fetus | 201528 (51.3) | 79156 (51.4) | 0.003 |
| Nulliparous | 191445 (48.7) | 62270 (40.5) | 0.167 |
| Prior stillbirth | 2706 (0.7) | 1330 (0.9) | 0.02 |
| Prior low birth weight infant | 6622 (1.7) | 2783 (1.8) | 0.009 |
| Prior caesarean section | 54847 (14.0) | 27142 (17.7) | 0.101 |
| IVF** | 6342 (2.4) | 2175 (2.4) | <0.001 |
| Suspected IUGR*** | 7689 (2.0) | 2420 (1.6) | 0.029 |
| Pre-pregnancy diabetes mellitus | 9243 (2.4) | 2684 (1.7) | 0.043 |
| Gestational diabetes | 48970 (12.5) | 16706 (10.9) | 0.05 |
| Pre-pregnancy hypertension | 2709 (0.7) | 909 (0.6) | 0.012 |
| Gestational hypertension | 33170 (8.4) | 11070 (7.2) | 0.047 |
| Chronic disease | 866 (0.2) | 446 (0.3) | 0.014 |
| Asthma | 2772 (0.7) | 995 (0.6) | 0.007 |
| Placental previa | 2664 (0.7) | 1236 (0.8) | 0.015 |
| Placental disorders (excl. previa) | 2816 (0.7) | 1008 (0.7) | 0.007 |
| Preeclampsia | 2421 (0.6) | 779 (0.5) | 0.015 |
| Gestational age at delivery |  |  |  |
| 20-27 |  |  | 0.076 |
| 28-33 | 1460 (0.4) | 1161 (0.8) |  |
| 34-36 | 3412 (0.9) | 1554 (1.0) |  |
| 37-38 | 26005 (6.6) | 11019 (7.2) |  |
| 39-41 | 122006 (31.1) | 50643 (32.9) |  |
| ≥42 | 235249 (59.9) | 87717 (57.0) |  |
| Smoking during pregnancy* | 31335 (8.0) | 14195 (9.2) | 0.045 |
| Alcohol use during pregnancy* | 3752 (1.0) | 1789 (1.2) | 0.02 |
| Drug use during pregnancy* | 11941 (3.0) | 5524 (3.6) | 0.031 |
| Congenital anomaly (any)**** | 1035 (0.3) | 474 (0.3) | 0.008 |

*Self-reported

**In-vitro-fertilization, information available only for pregnancies delivered in 2008/2009 onwards

*** Intrauterine growth restriction suspected on antenatal ultrasound (i.e., prior to delivery; antenatal ultrasound does not detect all growth-restricted fetuses theat are born as small-for-gestaional age)

**** Only among live births, diagnosed at birth
