# Supplementary material for: The association between pre-pregnancy body mass index and perinatal death and the role of gestational age at delivery
Source: PLoS One. 2022 Mar 23;17(3):e0264565. doi: 10.1371/journal.pone.0264565 (PMC8942230; doi:10.1371/journal.pone.0264565)
Supplement: S4 Table — (DOCX) [file pone.0264565.s005.docx]

S4 Table: Mediation analyses of BMI on perinatal death through gestational age at delivery after multiple imputation (n =10) of missing BMI values.

| **BMI Category** | **Underweight** | **Overweight** | **Obese** |
| --- | --- | --- | --- |
| Total effect (AOR-1) | 1.09 (0.93,1.28) | 1.20 (1.05,1.37) | 1.49 (1.2,1.86) |
| Natural direct | - | 1.13 (0.99,1.29) | 1.23 (0.99,1.54) |
| Natural indirect | - | 1.06 (0.93,1.21) | 1.21 (0.97,1.51) |
| Proportion mediated (indirect effect) |  | 31% | 47% |

Odds ratios were adjusted for chronic hypertension, smoking, substance/alcohol use, prior stillbirth, prior preterm birth, parity, maternal age, year of birth, chronic diseases, asthma
