# Supplementary material for: The association between pre-pregnancy body mass index and perinatal death and the role of gestational age at delivery
Source: PLoS One. 2022 Mar 23;17(3):e0264565. doi: 10.1371/journal.pone.0264565 (PMC8942230; doi:10.1371/journal.pone.0264565)
Supplement: S5 Table — (DOCX) [file pone.0264565.s006.docx]

S5 Table: Mediation analyses for stillbirth and neonatal death.

| **Outcome** | **BMI Category** | **Underweight** | **Overweight** | **Obese** |
| --- | --- | --- | --- | --- |
| Stillbirth | Total effect (AOR-1) | 1.03 (0.8,1.33) | 1.24 (1.08,1.43) | 1.66 (1.41,1.94) |
|  | Natural direct | 0.92 (0.73,1.16) | 1.13 (0.99,1.29) | 1.26 (1.09,1.46) |
|  | Natural indirect | 1.13 (1.02,1.25) | 1.10 (1.03,1.17) | 1.32 (1.22,1.42) |
|  | Proportion mediated (indirect effect) | - | 43% | 54% |
|  | | | | |
| Stillbirth antepartum | Total effect (AOR-1) | 1.00 (0.76,1.33) | 1.25 (1.07,1.47) | 1.64 (1.37,1.95) |
|  | Natural direct | 0.90 (0.68,1.17) | 1.15 (0.99,1.33) | 1.26 (1.06,1.49) |
|  | Natural indirect | 1.12 (1.02,1.24) | 1.09 (1.03,1.17) | 1.30 (1.2,1.41) |
|  | Proportion mediated (indirect effect) | - | 40.0% | 53% |
|  | | | | |
| Neonatal death | Total effect (AOR-1) | 1.31 (0.99,1.72) | 1.16 (0.97,1.38) | 1.41 (1.15,1.73) |
|  | Natural direct | 1.13 (0.89,1.44) | 1.02 (0.88,1.19) | 0.99 (0.83,1.18) |
|  | Natural indirect | 1.15 (1.00,1.33) | 1.13 (1.04,1.24) | 1.43 (1.28,1.59) |
|  | Proportion mediated (indirect effect) | - | 87% | - |

Odds ratios were adjusted for chronic hypertension, smoking, substance/alcohol use, prior stillbirth, prior preterm birth, parity, maternal age, year of birth, chronic diseases, asthma
