# Supplementary figures and images for: The association between pre-pregnancy body mass index and perinatal death and the role of gestational age at delivery
Source: PLoS One. 2022 Mar 23;17(3):e0264565. doi: 10.1371/journal.pone.0264565 (PMC8942230; doi:10.1371/journal.pone.0264565)

S1 Fig: Study population


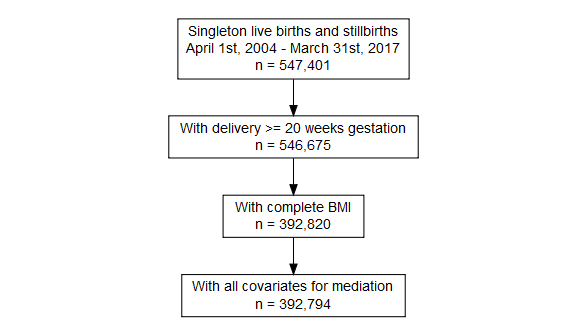

Supplement: S1 Fig — (DOCX) [file pone.0264565.s007.docx]
