# Supplementary material for: The association between pre-pregnancy body mass index and perinatal death and the role of gestational age at delivery
Source: PLoS One. 2022 Mar 23;17(3):e0264565. doi: 10.1371/journal.pone.0264565 (PMC8942230; doi:10.1371/journal.pone.0264565)
Supplement: S2 Fig — (C and L represent two sets of confounders: C confounders between the exposure and the outcomes; L confounders between the mediator and the outcome). (DOCX) [file pone.0264565.s008.docx]

S2 Fig: Directed acyclic graph representing assumed causal structure for causal mediation analysis.

(C and L represent two sets of confounders: C confounders between the exposure and the outcomes; L confounders between the mediator and the outcome).


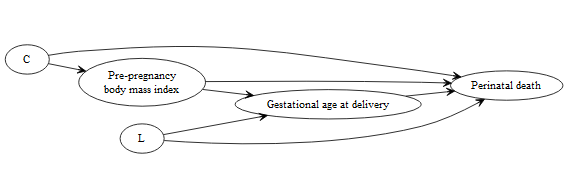


Mediation analyses in the text assume that all C and L are measured and adjusted for, and for the identification of natural direct and indirect effects that BMI does not affect any of the gestational age and perinatal death confounders (L). When this last assumption is violated, ‘interventional effects’ may still be estimated.
