# Supplementary material for: The association between pre-pregnancy body mass index and perinatal death and the role of gestational age at delivery
Source: PLoS One. 2022 Mar 23;17(3):e0264565. doi: 10.1371/journal.pone.0264565 (PMC8942230; doi:10.1371/journal.pone.0264565)
Supplement: S1 Appendix — (PDF) [file pone.0264565.s009.pdf]

Counterfactual mediation analysis

Let A, M, C and Y represent the exposure, mediator, confounders and outcome, respectively. The causal mediation effects can be defined as contrast of potential outcomes. Let M_a be the value that the mediator, possibly contrary to fact, be under exposure $A = a$, and let $Y_{a,m}$ me the value of the outcome, again possibly contract to fact, when $A = a$ and $M = m$. Then, we can define the natural direct effect as the contrast in Y between exposed vs not exposed assuming the mediator takes the value it would've in the non exposed, conditional on C. Likewise, we define the natural indirect effect as the contrast in Y in the exposed group, when the mediator takes the value it naturally would, vs what it would it take in the unexposed group, conditional on C. Namely, on the odds ratio scale we have (1-2):

$$\textbf{Natural direct effect} : OR^{NDE}_{a,a^*|C} = \frac{odds\{Y_{a,M_{a^*}} = 1|C\}}{odds\{Y_{a*,M_{a^*}} = 1|C\}}$$

and

$$\textbf{Natural indirect effect} : OR^{IDE}_{a,a^*|C} = \frac{odds\{Y_{a,M_a} = 1|C\}}{odds\{Y_{a,M_{a^*}} = 1|C\}}$$

Causal interpretation of these natural effects requires four assumptions:

1. No unmeasured confounding between A and Y
2. No unmeasured confounding between M and Y
3. No unmeasured confounding between A and M
4. No confounder of M and Y is effected by A

In our text, A=pre-pregnancy BMI, M = gestational age at delivery, C is the set of confounders listed in the main text, and Y = perinatal death. Note also that our outcome is very rare ($< 1\%$) and therefore the above odds ratios approximate the risk ratios.

Mediation analysis in presence of exposure induced mediator outcome confounding

As discussed in the main text, it is likely that in our example (as in many other applied situations) there is likely to be some confounder, L of gestational age and perinatal death that is also caused by pre-pregnancy BMI (e.g. pre-pregnancy diabetes) and therefore assumption 4 above may be violated.

In this case, Vanderweele & Vansteelandt (3-5) have shown that we can still estimate 'interventional' versions of the direct and indirect effects listed above.

In our case, thee above natural direct and indirect effects correspond for each *individual* to setting the gestational age at delivery of those with high BMI to values of what it would be if they were normal BMI. On the other hand, the interventional versions of these effects correspond to the direct and indirect effects if those with high BMI had there gestational age randomly drawn from the distribution of gestational ages of those with normal BMI. That is, we now have effects based on *population* rather than *individual* counterfactuals. In our case, corresponding to shifting the gestational age at delivery distribution of obese women to match the gestational age delivery distribution of those of normal BMI.

Formally, assuming the above notation and letting $M \sim f_{a|c}$, (i.e. the distribution of M amongst those with $A = a$, conditional on $C = c$) and $G_{a|c}$ be a random draw from f, then (3-4)

$$\textbf{Interventional direct effect} : OR^{IDE}_{a,G_{a^*|c}|C} = \frac{odds\{Y_{a,G_{a^*|c}} = 1|C\}}{odds\{Y_{a*,G_{a^*|c}} = 1|C\}}$$

and

$$\textbf{Interventional indirect effect} : OR^{IIE}_{a,G_{a^*|c}|C} = \frac{odds\{Y_{a,G_{a|c}} = 1|C\}}{odds\{Y_{a,G_{a^*|c}} = 1|C\}}$$

These effects can be identified and unbiased assuming that L is adjusted for. Our second set of adjusted odds ratios in the main text correspond to these effects where $L = \{$**pre-pregnancy diabetes, congenital anomolies**$\}$. Our analyses were broadly conistent after adjustment for L, but as mentioned, there are additional potential variables in L that could effect our results.

Estimation of effects with multiple versions of the treatment

When studying exposures like obesity, there is much debate on how results should be interrupted given that reducing obesity may be done many ways (i.e. that the treatment is not well defined) and therefore the idea of a 'causal effect' of obesity (or similar exposures) cannot be estimated (6-11). In cases such as this, we can still estimate the effect of no one being obese by changing the determinants of obesity to reflect the determinants of those non-obese in the population (see 3.2 in 11). Of course this does not correspond to a specific intervention on obesity, but the estimate of this 'effect' is function of the population distribution of obesity (ref). By using a potential outcomes framework in this study we have tried to be clear about the assumptions required for our analyses, and the corresponding limitations. Exposures like obesity will continue to be studied in clinical and epidemiological studies regardless of these issues, and we hope by being explicit about the limitations the results can be interpreted appropriately. Further, in this example, our focus was on explaining pathways in the general population of obese women and generating potential explanations.

Although no 'fix-all' intervention exists for extending pregnancies, a hypothetical intervention here is plausible. By beginning the messy process of untangling such pathways we can begin to further refine questions, and eventually tailor appropriate obstetric responses.

References

1. VanderWeele TJ. Mediation Analysis: A Practitioners Guide. Annu Rev Public Health. 2016 Mar 18;37(1):1732.

2. Lange T, Vansteelandt S, Bekaert M. A simple unified approach for estimating natural direct and indirect effects. Am J Epidemiol. 2012 Aug;176(3):1905.

3. Vanderweele TJ, Vansteelandt S, Robins JM. Effect decomposition in the presence of an exposure-induced mediator-outcome confounder. Epidemiology. 2014 Mar;25(2):3006.

4. Vansteelandt S, Vanderweele TJ. Natural Direct and Indirect Effects on the Exposed: Effect Decomposition under Weaker Assumptions. Biometrics. 2012 Dec;68(4):101927.

5. Vansteelandt S, Daniel RM. Interventional Effects for Mediation Analysis with Multiple Mediators. Epidemiology. 2017 Mar 1;28(2):25865.

6. VanderWeele TJ, Hernan MA. Causal inference under multiple versions of treatment. J Causal Inference. 2013 Jun 5;1(1):120.

7. Hernn MA, Vanderweele TJ. Compound treatments and transportability of causal inference. Epidemiology. 2011 May;22(3):36877.

8. Laan M Van Der, TH-AJ of, 2005 undefined. van der Laan et al. respond to Hypothetical interventions to define causal effects.

9. Pearl J. On the consistency rule in causal inference: Axiom, definition, assumption, or theorem? Epidemiology. 2010 Nov;21(6):8725.

10. Hernn MA, Taubman SL. Does obesity shorten life? The importance of well-defined interventions to answer causal questions. Int J Obes. 2008;32:S814.

11. Hernn MA, Robins JM (2020). Causal Inference: What If. Boca Raton: Chapman & Hall/CRC.
